# Effects of Prey Presence and Scale on Bobcat Resource Selection during Winter

**DOI:** 10.1371/journal.pone.0143347

**Published:** 2015-11-18

**Authors:** Florent Bled, Savanna Summers, Deborah Martell, Tyler R. Petroelje, Dean E. Beyer, Jerrold L. Belant

**Affiliations:** 1 Carnivore Ecology Laboratory, Forest and Wildlife Research Center, Mississippi State University, Mississippi, United States of America; 2 Wildlife Division, Michigan Department of Natural Resources, Marquette, Michigan, United States of America; University of Sydney, AUSTRALIA

## Abstract

Factors relevant to resource selection in carnivores may vary across spatial and temporal scales, both in magnitude and rank. Understanding relationships among carnivore occupancy, prey presence, and habitat characteristics, as well as their interactions across multiple scales, is necessary to improve our understanding of resource selection and predict population changes. We used a multi-scale dynamic hierarchical co-occurrence model with camera data to study bobcat and snowshoe hare occupancy in the Upper Peninsula of Michigan during winter 2012–2013. Bobcat presence was influenced at the local scale by snowshoe hare presence, and by road density at the local and larger scale when hare were absent. Hare distribution was related primarily to vegetation cover types, and detectability varied in space and time. Bobcat occupancy dynamics were influenced by different factors depending on the spatial scale considered and the resource availability context. Moreover, considering observed co-occurrence, we suggest that bobcat presence had a greater effect on hare occupancy than hare presence on bobcat occupancy. Our results highlight the importance of studying carnivore distributions in the context of predator-prey relationships and its interactions with environmental covariates at multiple spatial scales. Our approach can be applied to other carnivore species to provide insights beneficial for management and conservation.

## Introduction

Habitat selection refers to the process by which an animal, through a series of hierarchical behavioral responses might choose to disproportionally use some areas of its habitat [1]. In a heterogeneous environment, this process may influence the survival and fitness of individuals making this choice. Habitat use, or the way an animal uses the collection of conditions and resources in a habitat, is the result of this selection. Multiple resources and environmental factors (e.g. cover, food, or the presence of predators or conspecifics) can influence habitat selection and result in variations of habitat use [2][3]. Consequently, because the factors relevant to this selection process may vary across spatial or temporal scales in magnitude and rank [4][5][6][7], habitat use will be scale-dependent. For example, while a forest might be considered used by a predator at a large scale, at a finer scale some parts might be preferentially used for refugia or hunting, while other areas might not be used at all. Some of the factors influencing spatio-temporal habitat selection include season [8], intraspecific interactions [9], breeding behaviors [10], landscape attributes (e.g., land cover heterogeneity) [11] and fragmentation or human development [12][13]. Moreover, the relative importance of factors influencing habitat selection and subsequent use may also vary depending on the scale considered. Animals can select different resources at different scales [14][15][16]. For example, Canada lynx (*Lynx Canadensis*) selected dens based on topography at a greater spatial scale, followed by forest seral stage and horizontal cover/log volume at increasingly finer scales [17]. Distributions analyses should consider the effects of scale when estimating the effects of factors underlying habitat selection.

For carnivores, resources including landscape features and characteristics as well as prey availability will impact habitat use [18]. For example, forest cover types, and especially vegetation cover was identified as one of the factors that influenced habitat selection by bobcats (*Lynx rufus*) [19] and grizzly bears (*Ursus arctos*) [20]. However, more important than this factor was prey availability. Murray, Boutin & O'Donoghue (1994) [21] found that lynx (*Lynx canadensis*) and coyote (*Canis latrans*) occupancy shifted with variation in availability of snowshoe hare. Landscape features such as road density and hydrogeography can complement the set of factors involved in carnivore habitat selection. While the presence of a developed road network can have a negative effect [22], linear features such as smaller roads or trails can also be used as travel corridors ([23]; e.g., gray wolves (*C*. *lupus*) [24] or cougars (*Puma concolor*) [25]. The repartition (both absolute and relatively to each other) of these factors will vary spatially and as a function of the scale considered.

Since one of the main factors responsible for carnivore habitat selection is prey availability, to accurately predict carnivore habitat use, we need to account for their prey distribution, as well as the factors affecting this prey habitat use. For several medium-sized carnivore species, including bobcats, the target species of our study, snowshoe hare (*Lepus americanus*) is an important prey species [26]. Snowshoe hare prefers dense understory [27] and to forage near cover [28], possibly in response to the high predation pressure that the species is facing. In this context, cover types and proportions is an essential factor to consider when modeling snowshoe hare habitat use.

We addressed habitat use of bobcats (*Lynx rufus*) in the Upper Peninsula of Michigan during winter. Specifically, we estimated how bobcat occupancy was influenced by landscape factors as well as by snowshoe hare habitat use. In turn, we considered cover types structure to model hare occupancy. During winter, most bobcat activity is associated with foraging [29]; consequently, bobcat occupancy should be linked to prey availability [2][21][30]. Moreover, linear features such as roads and streams may also influence bobcat occupancy [22] because of learned behavior to avoid high activity roads due to risk or use as travel corridors. Our objective was to determine the factors governing distribution of bobcat and its prey. We were also interested in how the factors impacting bobcat occupancy could vary depending on the spatial scale at which they are considered. Because snowshoe hare is an important prey of bobcats during winter, we predicted bobcat occupancy would increase in areas with greater snowshoe hare occupancy. We predicted that bobcat occupancy probability would be positively correlated with the hydrogeography characteristics of our survey area (i.e. extent of rivers and streams). We also hypothesized bobcats would avoid roads at fine and coarse spatial scales, due to associated risk. Finally, we expected a positive association between hare occupancy and dense vegetative cover types. Thanks to a novel multi-scale hierarchical model we have been able to assess these hypotheses and provide directions to improve our understanding of habitat selection and use by a medium sized carnivore.

## Material and Methods

### Study design

We conducted the study in a 400 km^2^ square area, north of the Michigamme Reservoir in the Upper Peninsula of Michigan (46°15' N, 88°14' W). Land cover included deciduous forests (38%), woody wetlands (29%), mixed forests (13%), conifer forests (6%), open water (4%), grassland/herbaceous (3%), developed (3%), and other (3%). Dominant tree species include sugar maple (*Acer saccharum*) and trembling aspen (*Populus tremuloides*) in upland deciduous forests, black spruce (*Picea mariana*) in lowland coniferous forests, and red pine (*Pinus resinosa*) in plantations. Average annual snowfall in the study area is about 180 cm and average annual rainfall is about 69 cm. State and US highways border the study area with secondary roads interspersed throughout. Land within the study area is state, commercial forest association, or privately owned.

We divided the study area into 64 2.5 x 2.5 km cells, and selected a station in each cell that we considered suitable bobcat habitat, following Stricker et al. [29]. At each station, we constructed a circular barrier 0.7–1.0 m high and containing 4 entrances, then baited stations using vehicle- or hunter-killed white-tailed deer (*Odocoileus virginianus*) or beaver (*Castor canadensis*) carcasses wired to a central tree. We applied a commercial skunk-based lure to the central tree about 1.7 m above ground. At each station, we attached a remote camera (Bushnell Infrared Trophy Cameras; Bushnell Outdoor Products, Overland Park, Kansas, USA) to a tree, 70 to 100 cm above ground and positioned to capture all animal activity within the station. We programmed cameras to obtain images of animals with a 5-minute delay between detections. We conducted an 11 week survey from December 2012 to February 2013 with visits to each station every 7 days. We replaced image cards and batteries each visit and added bait and lure as necessary. We recorded the date and time of each image containing bobcat or hare detected and summarized by week and species the number of days the species was detected, and the number of days each camera operated (i.e., number of sampling replicates). In this context, we assumed occupancy to be constant during each week, and used each day in a week as a temporal replicate. Therefore, most cells received 7 temporal replicates (i.e. days) each week.

### Covariates

Using remote-sensing data, we extracted covariates for each cell in our survey grid. To model bobcat occupancy, we used the following covariates: road and water densities at the scale of the cell and of the first-order neighborhood (defined as the eight cells surrounding the target cell), and distance from the station to the nearest water source. Full neighborhood information for cells in the border of the study area was provided via a one-cell buffer area surrounding our target region. We obtained river, stream and road data from Topologically Integrated Geographic Encoding and Referencing system files [31]. We modeled snowshoe hare occupancy as a function of vegetation cover type. We used 2011 National Landcover Data (30-m resolution; [32]) that we reclassified from 15 original landcovers to 9 (Table 1). For each cell, we then estimated the percentage of the cell covered by each vegetation cover type. Each covariate was scaled and centered for analyses, and no strong or moderate multicollinearity was detected [33]. The range of each covariate, once scaled, is presented in S1 Table.

**Table 1:** Land cover designations as defined in the 2011 national land cover database with percentage land cover within study area, Iron County, Upper Peninsula Michigan (from [32]).

Land cover	Description
Deciduous forest excluding aspen (DEA)	Areas dominated by trees (excluding aspen) generally greater than 5 meters tall, and greater than 20% of total vegetation cover. More than 75 percent of the tree species shed foliage simultaneously in response to seasonal change.
Aspen forest	Areas where aspens are present and dominated by trees generally greater than 5 meters tall, and greater than 20% of total vegetation cover. More than 75 percent of the tree species shed foliage simultaneously in response to seasonal change.
Woody wetland (WW)	Areas where forest or shrub land vegetation accounts for greater than 20 percent of vegetative cover and the soil or substrate is periodically saturated with or covered with water.
Mixed forest	Areas dominated by trees generally greater than 5 meters tall, and greater than 20% of total vegetation cover. Neither deciduous nor evergreen species are greater than 75 percent of total tree cover.
Evergreen forest	Areas dominated by trees generally greater than 5 meters tall, and greater than 20% of total vegetation cover. More than 75 percent of the tree species maintain their leaves all year. Canopy is never without green foliage.
Open water	Areas of open water, generally with less than 25% cover or vegetation or soil
Grassland/herbaceous	Areas dominated by grammanoid or herbaceous vegetation, generally greater than 80% of total vegetation. These areas are not subject to intensive management such as tilling, but can be utilized for grazing.
Emergent herbaceous wetland (EHW)	Areas where perennial herbaceous vegetation accounts for greater than 80 percent of vegetative cover and the soil or substrate is periodically saturated with or covered with water.
Shrub/scrub	Areas dominated by shrubs; less than 5 meters tall with shrub canopy typically greater than 20% of total vegetation. This class includes true shrubs, young trees in an early successional stage or trees stunted from environmental conditions.
Unsuitable (i.e., urban, barren, pasture, agriculture)	All other areas modified by agriculture or developed land use practices such as farmed row crops, pastures, roads, and structures.

### Statistical analysis

We estimated bobcat and snowshoe hare occupancies using a hierarchical co-occurrence model [34]. We first modeled snowshoe hare occupancy $Z_{i,t}^{H}$ in cell *i* during week *t* as following a Bernoulli distribution with a mean $\mu_{i,t}^{H}$, that we modeled as a linear function of landcover covariates on the logit scale, such as:

$$Z_{i,t}^{H}{}_{\;}\sim Bern\left(\mu_{i,t}^{H}\right)$$

$$\begin{array}{l} logit\left(\mu_{i,t}^{H}\right)=b^{H}+\beta_{Aspen}Aspen_{i}+\beta_{DEA}DEA_{i}+\beta_{Evergreen}Evergreen_{i}+\beta_{Mixed}Mixed_{i}\\ +\beta_{Shrub}Shrub_{i}+\beta_{Grassland}Grassland_{i}+\beta_{WW}WW_{i}+\beta_{EHW}EHW_{i}+\beta_{Unsuitable}Unsuitable_{i}\\ +\varepsilon_{i,t}^{H} \end{array}$$

where *b* ^*H*^ is the intercept and *β*_*x*_ corresponds to the ‘x’ vegetation type specific slope. The intercept was given a flat prior, while the slopes for cover type covariates were given non-informative Normal priors with mean 0 and variance 100. We accounted for any residual spatial and time variations using the random effect $\varepsilon_{i,t}^{H}$, following a normal distribution such as:

$$\varepsilon_{i,t}^{H}\sim Normal\left(0,\sigma_{hare}^{2}\right)$$

Similarly, bobcat occupancy $Z_{i,t}^{B}$ followed a Bernoulli distribution with a mean $\mu_{i,t}^{B}$. In turn, $\mu_{i,t}^{B}$ is defined conditionally on hare presence, as we expected bobcat occupancy probability to be greater when its prey is present.

$$\mu_{i,t}^{B}=Z_{i,t}^{H}\;.\;\mu_{1\;i,t}^{B}+\left(1-Z_{i,t}^{H}\right).\;\mu_{0\;i,t}^{B}$$

where $\mu_{1\;i,t}^{B}$ and $\mu_{0\;i,t}^{B}$ represent bobcat occupancy when hare is present and absent, respectively. We then modeled those probabilities as a linear function of water and road covariates on the logit scale:

$$logit\left(\mu_{1\;i,t}^{B}\right)=b_{1}^{B}+\alpha_{1\;road}D_{road\left(c\right)\;i}+\alpha_{1\;water}D_{water\left(c\right)\;i}+\beta_{1\;road}D_{road\left(n\right)\;i}+\beta_{1\;water}D_{water\left(n\right)\;i}+\gamma_{1}d_{i}+\varepsilon_{i,t}^{B}$$

$$logit\left(\mu_{0\;i,t}^{B}\right)=b_{0}^{B}+\alpha_{0\;road}D_{road\left(c\right)\;i}+\alpha_{0\;water}D_{water\left(c\right)\;i}+\beta_{0\;road}D_{road\left(n\right)\;i}+\beta_{0\;water}D_{water\left(n\right)\;i}+\gamma_{0}d_{i}+\varepsilon_{i,t}^{B}$$

Thus, $b_{1}^{B}$ and $b_{0}^{B}$ are intercepts for bobcat occupancy probabilities when hare are present and absent, respectively. When hare are present, slopes for the cell road and water densities (*D*_road(c)i_ and *D*_water(c)i_), neighborhood road and water densities (*D*_road(n)i_ and *D*_water(n)i_), and distance to nearest water source (*d*_*i*_) are represented by *α*_1 road_, *α*_1 water_, *β*_1 road_, *β*_1 water_ and *γ*_1_, respectively. When hare are absent, *α*_0 road_, *α*_0 water_, *β*_0 road_, *β*_0 water_ and *γ*_0_ are the corresponding slopes. Intercepts were given flat priors, while slopes for road and water covariates were given non-informative Normal priors with mean 0 and variance 10000. Finally, $\varepsilon_{1\;i,t}^{B}$ and $\varepsilon_{0\;i,t}^{B}$ are random cell-week effects. This formulation allows bobcat occupancy, as well as its relationship to the covariates, to vary with hare presence.

We used a hierarchical approach to model bobcat and snowshoe hare and their potential ecological determinants independently of the effects of imperfect detection and sampling [35]. We modeled bobcat and snowshoe hare detections, $Y_{i,t}^{B}$ and $Y_{i,t}^{H}$, respectively, in cell *i* during week *t* as following a binomial distribution such that:

$$Y_{i,t}^{B}\sim Binomial\left(K_{i,t}\;,\;p_{i}^{B}.Z_{i,t}^{B}\right)$$

$$Y_{i,t}^{H}\sim Binomial\left(K_{i,t}\;,\;p_{i,t}^{H}.Z_{i,t}^{H}\right)$$

where $p_{i,t}^{H}$ is the daily hare detection probability during week *t* in cell *i*, and $\;p_{i}^{B}$ is the daily bobcat detection probability in cell *i*. *K*_*i*,*t*_ indicates the number of days in week *t* during which the camera in cell *i* was operating, and therefore corresponds to 7 temporal replicates for most cell-week combinations.

### Implementation

We implemented the model using program WinBUGS [36], called from R (v.3.0.1, [37]) with the package R2WinBUGS [38] (S1 File, corresponding data available in S1 Dataset). We ran three chains using non-informative priors, for 100,000 iterations after a 300,000 iteration burn-in period (to insure convergence) with a thinning of 10. We monitored convergence by visual inspection of the MCMC chains and using the Gelman-Rubin convergence statistic $\hat{R}$ [39].

Model selection was done following a two-step process: 1) selecting the general observation process modeling structure and 2) selecting the relevant set of covariates used in the ecological process part of the model. First, we determined a suitable general model structure for our observation process by assessing goodness-of-fit based on Bayesian p-values both for bobcat and hare weekly total detection [40]. Our original model considered daily bobcat and hare detection probabilities (*p* ^*B*^ and *p* ^*H*^, respectively) to be constant over time and space since we did not expect variation in our monitoring using cameras. However, fitting this initial model resulted in poor goodness-of-fit (Bayesian p-values [41] of 0.00 for hare and 0.01for bobcat; S1 Fig). We therefore revised the model to allow the daily bobcat detection probability $p_{i}^{B}$ to vary across space and the daily hare detection probability $p_{i,t}^{H}$ across both space and time (i.e. between weeks), resulting in satisfactory goodness-of-fit (Bayesian p-values of 0.77 for hare and 0.38 for bobcat; S2 Fig). We tested a final model where bobcat and hare detection probabilities varied across space and time, but the improvement in goodness-of-fit for bobcat did not warrant the additional parameters, with Bayesian p-value of 0.77 and 0.69 for hare and bobcat, respectively. Secondly, to determine which covariates were important to explain bobcat and hare occupancy, we specified a set of latent indicator variables *w*_*k*_ (one for each model effect *k*) [41]. By imposing a Bernoulli prior on each indicator variable, we can dictate the probability of each set of model covariates, and therefore their probability of being included in the final model [42] [35]. Consider the set of later indicator variables

$$w_{k}={\{}_{0\text{ if not}}^{1\text{ if covariate }k\text{ is included in the model}}$$

with prior distributions:

$$w_{k}\sim Bern\left(0.5\right)$$

assumed to be mutually independent. We can then reformulate the occupancy probabilities definitions such as:

$$logit\left(\mu_{1\;i,t}^{B}\right)=b_{1}^{B}+w_{1}\alpha_{1\;road}D_{road\left(c\right)\;i}+w_{2}\alpha_{1\;water}D_{water\left(c\right)\;i}+w_{3}\beta_{1\;road}D_{road\left(n\right)\;i}+w_{4}\beta_{1\;water}D_{water\left(n\right)\;i}+w_{5}\gamma_{1}d_{i}+\varepsilon_{i,t}^{B}$$

$$logit\left(\mu_{0\;i,t}^{B}\right)=b_{0}^{B}+w_{6}\alpha_{0\;road}D_{road\left(c\right)\;i}+w_{7}\alpha_{0\;water}D_{water\left(c\right)\;i}+w_{8}\beta_{0\;road}D_{road\left(n\right)\;i}+w_{9}\beta_{0\;water}D_{water\left(n\right)\;i}+w_{10}\gamma_{0}d_{i}+\varepsilon_{i,t}^{B}$$

$$\begin{array}{l} logit\left(\mu_{i,t}^{H}\right)=b^{H}+w_{11}\beta_{Aspen}Aspen_{i}+w_{12}\beta_{DEA}DEA_{i}+w_{13}\beta_{Evergreen}Evergreen_{i}+w_{14}\beta_{Mixed}Mixed_{i}+w_{15}\beta_{Shrub}Shrub_{i}\\ +w_{16}\beta_{Grassland}Grassland_{i}+w_{17}\beta_{WW}WW_{i}+w_{18}\beta_{EHW}EHW_{i}+w_{19}\beta_{Unsuitable}Unsuitable_{i}+\varepsilon_{i,t}^{H} \end{array}$$

Finally, based on the posterior distribution each *w*_*k*_, we estimated the likelihood of each corresponding *k* covariate to be included in the final model.

To provide a simple summary of co-occurrence probabilities, we estimated a posterior co-occurrence contingency table indicating how many times across cells and weeks bobcat and hare were each present, present together, or neither present. Based on this posterior contingency table, we then estimated the probability that the proportion of cells occupied by bobcats when hares were present was greater than, equal to, or less than the proportion of cells occupied by bobcats when hares were absent. Let *y*_*1*_ and *y*_*2*_ be the number of cells occupied by bobcats when hare is present or absent, respectively. Let *n*_*1*_ and *n*_*2*_ be the total number of cells occupied or not occupied by hares, respectively. Then, the number of cells occupied by bobcats depending on hare occupancy can be defined as:

$$y_{1}\;\sim \;Binom\left(n_{1},\theta_{1}\right)$$

$$y_{2}\;\sim \;Binom\left(n_{2},\theta_{2}\right)$$

with *θ*_*1*_ and *θ*_*2*_ representing the proportion of sites that are occupied by bobcat when hare is present and absent, respectively. Assuming Beta(1,1) priors (equivalent to a uniform distribution) on the proportions *θ*_*1*_ and *θ*_*2*_, and given the conjugacy of the beta for the binomial, we have:

$$p(\theta_{1}|y_{1},\;n_{1})=\;Beta\left(\theta_{1}|y_{1}+1,n_{1}-y_{1}+1\right)$$

$$p(\theta_{2}|y_{2},\;n_{2})=\;Beta\left(\theta_{2}|y_{2}+1,n_{2}-y_{2}+1\right)$$

From there, we can estimate by simulation the posterior density of *δ = θ*_*1*_ *-θ*_*2*_, the probability that *θ*_*1*_ is greater than *θ*_*2*_, i.e. the probability that cells are more likely to be occupied by bobcats when hare are present (compared to when hare are absent). This simulation is done by taking M = 10000 samples from the joint posterior

$$p(\theta_{1},\theta_{2}\left|y_{1},y_{2},\;n_{1},n_{2})\;=\;p(\theta_{1}\right|y_{1},\;n_{1})\;\times \;p(\theta_{2}|y_{2},\;n_{2})$$

and then use the approximation

$$\text{Pr}(\theta_{1}>\theta_{2})\approx \frac{1}{M}\sum_{M}^{m=1}I(\theta_{1}^{\left(m\right)}>\theta_{2}^{\left(m\right)})$$

where $I\left(\theta_{1}^{\left(m\right)}>\theta_{2}^{\left(m\right)}\right)=1$, if $\theta_{1}^{\left(m\right)}$ is greater than $\theta_{2}^{\left(m\right)}$, and 0 otherwise. More details concerning this approach can be found following Carpenter [43] and Gelman [44] [39]. We conducted a similar assessment of hare occupancy response to bobcat presence/absence.

### Ethics statement

This research was conducted with approval from The Mississippi State University Institutional Animal Care and Use Committee, Animal Ethics Committee, Animal Ethics Permit number CMLR/304/13/QLD GOVT Protocol Number 09–004, was carried out in the field according to the conditions of Michigan Department of Natural Resources scientific collector permit number 1376.

## Results

### Detectabilities

**Fig 1:**
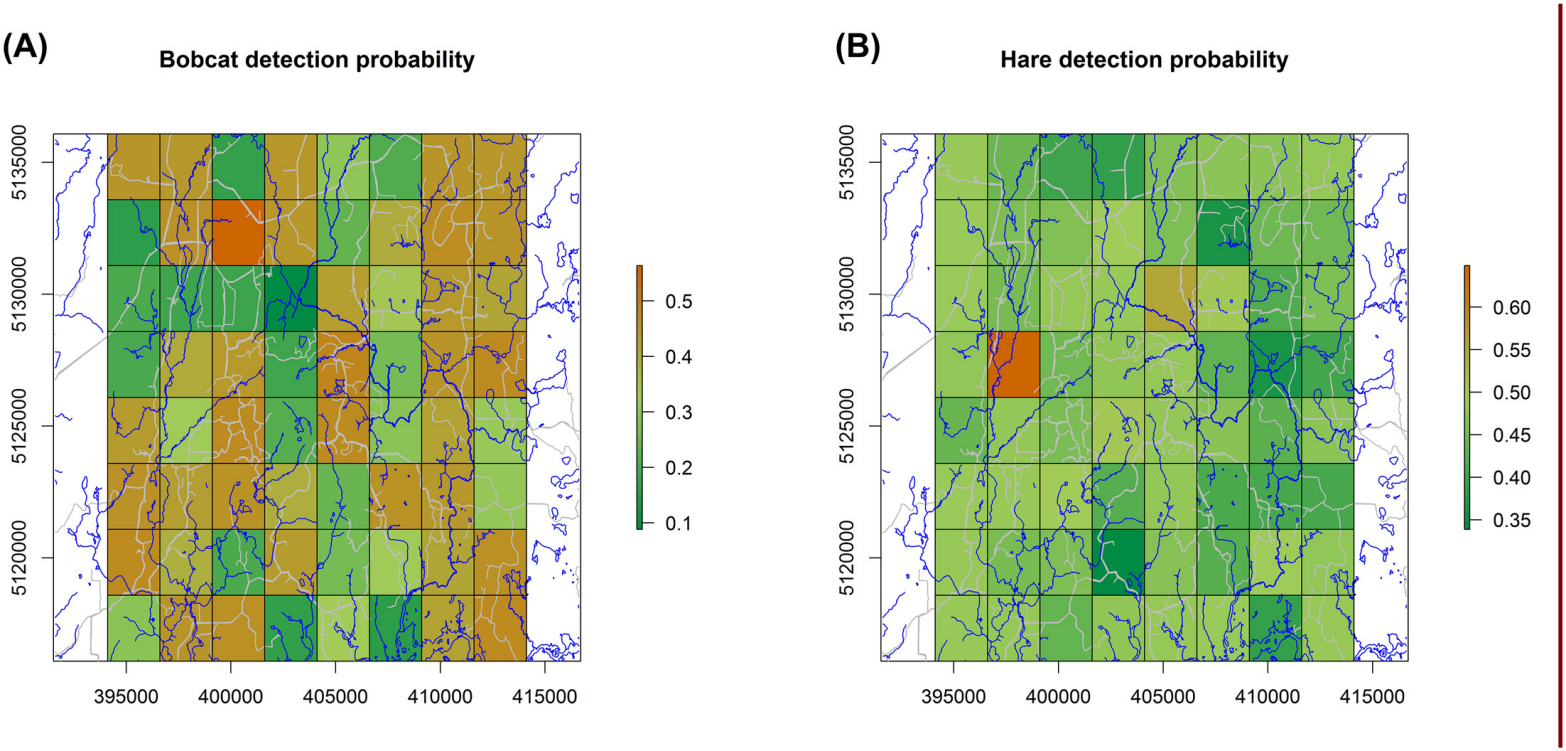
Bobcat (A) and snowshoe hare (B) detection probabilities averaged over weeks, Upper Peninsula Michigan, December 2012–February 2013. Roads and streams are represented by grey and blue lines, respectively.

Mean hare detection probability was 0.46 ± 0.28 (mean ± standard deviation) averaged across weeks and cells) and mean bobcat detection probability was 0.36 ± 0.27 (averaged across cells). As expected, following model selection, both bobcat and hare detection probabilities vary across space (Fig 1).

### Hare occupancy

**Fig 2:**
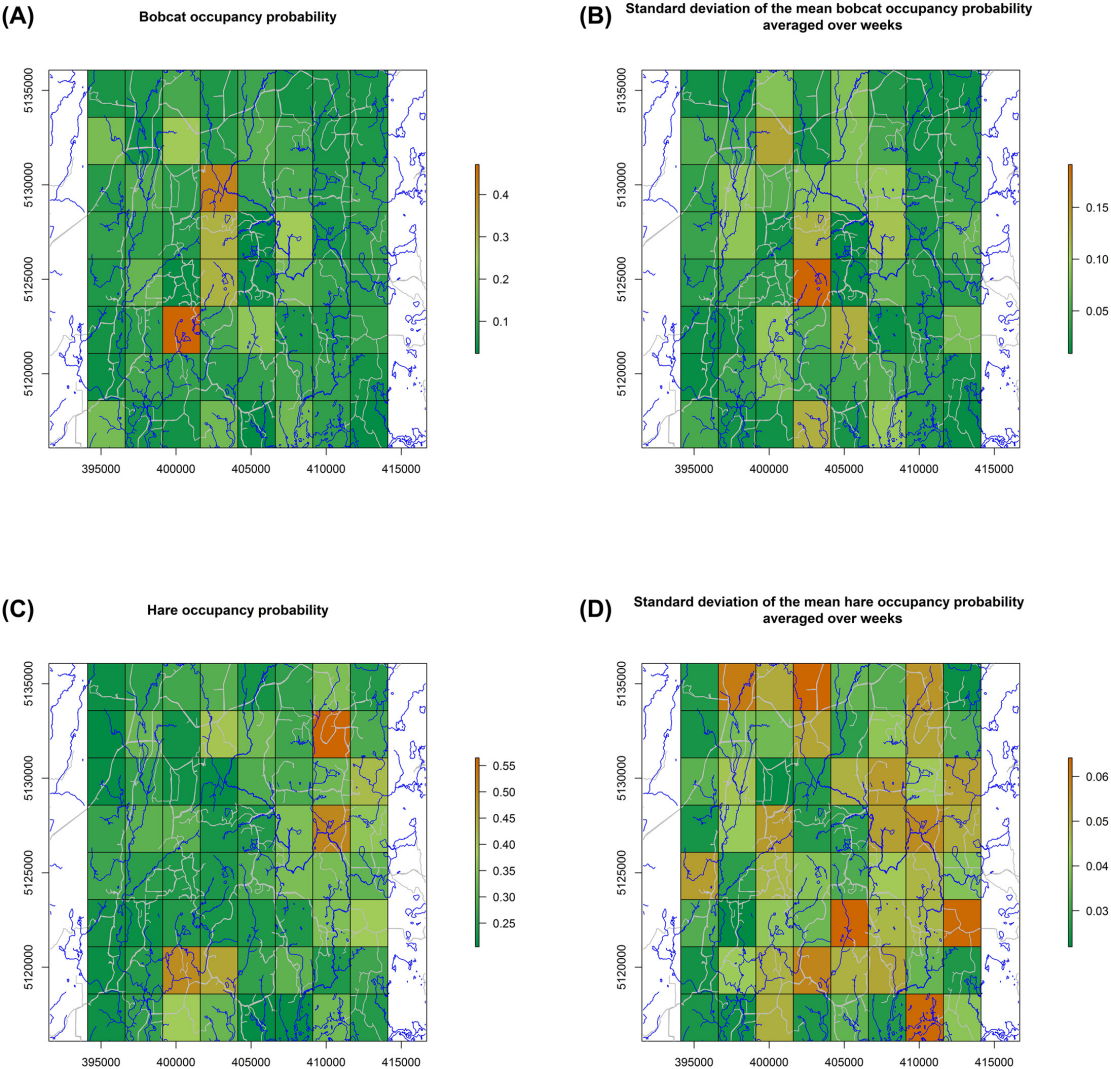
Bobcat occupancy probability (and corresponding standard deviations) (A & B) and snowshoe hare occupancy (and corresponding standard deviations) (C & D) averaged over weeks for each cell, Upper Peninsula Michigan, December 2012–February 2013. Roads and streams are represented by grey and blue lines, respectively.

Overall mean hare occupancy probability (averaged over weeks and cells) was 0.29± 0.15 with greatest occupancy in the northeast and southwest portions of the study area (Fig 2). While overall mean hare occupancy probability did not vary greatly over time, cell specific probabilities varied in space and time. Hare occupancy was positively correlated to grassland cover type, and had a probability of being selected in the model of 0.95 (Table 2).

**Table 2:** Intercepts and slopes on the logit scale for the selected bobcat-snowshoe hare hierarchical co-occurrence model in Upper Peninsula Michigan, during December 2012—February 2013. Model selection probability corresponds to the percentage of iterations in which the corresponding covariate was selected to be part of the model. Corresponding parameter values are only indicated for model selection probabilities higher than 0.5.

Response variable	Parameter	Scale	Mean	95% Credible Interval	Model selection probability
**Bobcat occupancy probability (when hare present)**	Intercept ($b_{1}^{B}$)		-3.2	[-4.18; -2.04]	NA
	Road density (*α*_1 road_)	Cell	-		0.04
	Road density (*β*_1 road_)	Neighborhood	-		0.01
	Water density (*α*_1 water_)	Cell	-		0.03
	Water density (*β*_1 water_)	Neighborhood	-		0
	Distance to water (*γ*_1_)		-		0
**Bobcat occupancy probability (when hare absent)**	Intercept ($b_{0}^{B}$)		-2.82	[-3.44; -2.24]	NA
	Road density (*α*_0 road_)	Cell	-1.04	[-1.69; -0.43]	1
	Road density (*β*_0 road_)	Neighborhood	0.83	[0.31;1.40]	1
	Water density (*α*_0 water_)	Cell	-		0
	Water density (*β*_0 water_)	Neighborhood	-		0
	Distance to water (*γ*_0_)		-		0
**Hare occupancy probability**	Intercept (*b* ^*H*^)		-1.03	[-1.39; -0.77]	NA
	*β*_*DEA*_	Cell	-		0
	*β*_*Aspen*_	Cell	-		0
	*β*_*Mixed*_	Cell	-		0
	*β*_*Shrub*_	Cell	-		0.07
	*β*_*WW*_	Cell	-		0.01
	*β*_*Unsuitable*_	Cell	-		0.16
	*β*_*Evergreen*_	Cell	-		0.07
	*β*_*Grassland*_	Cell	0.41	[0.18; 0.69]	0.95
	*β*_*EHW*_	Cell	-		0
**Bobcat detection probability**	Detection probability ($\;p_{i}^{B}$)	Cell	NA		NA
**Hare detection probability**	Detection probability ($p_{i,t}^{H}$)	Cell + week	NA		NA

‘-’: parameter not selected in the final model, ‘NA’: non-applicable

### Bobcat occupancy

Overall mean bobcat occupancy probability (averaged over weeks and cells) was 0.11 ± 0.14. Occupancy was greatest near the center of the study area and occupancy of most cells varied little over time (Fig 2). When hares were absent, bobcat occupancy probability was negatively correlated with road density at the cell scale, and positively correlated at the neighborhood scale (Table 2), and both of these parameters had a probability of being selected in the model equal to 1. When hares were present, bobcat occupancy probability was not influenced by other covariates and mean bobcat occupancy (averaged over weeks and cells) appeared lower when hares were present (0.07 ± 0.07 against 0.12 ± 0.16 with hares absent).

### Co-occurrence

**Fig 3:**
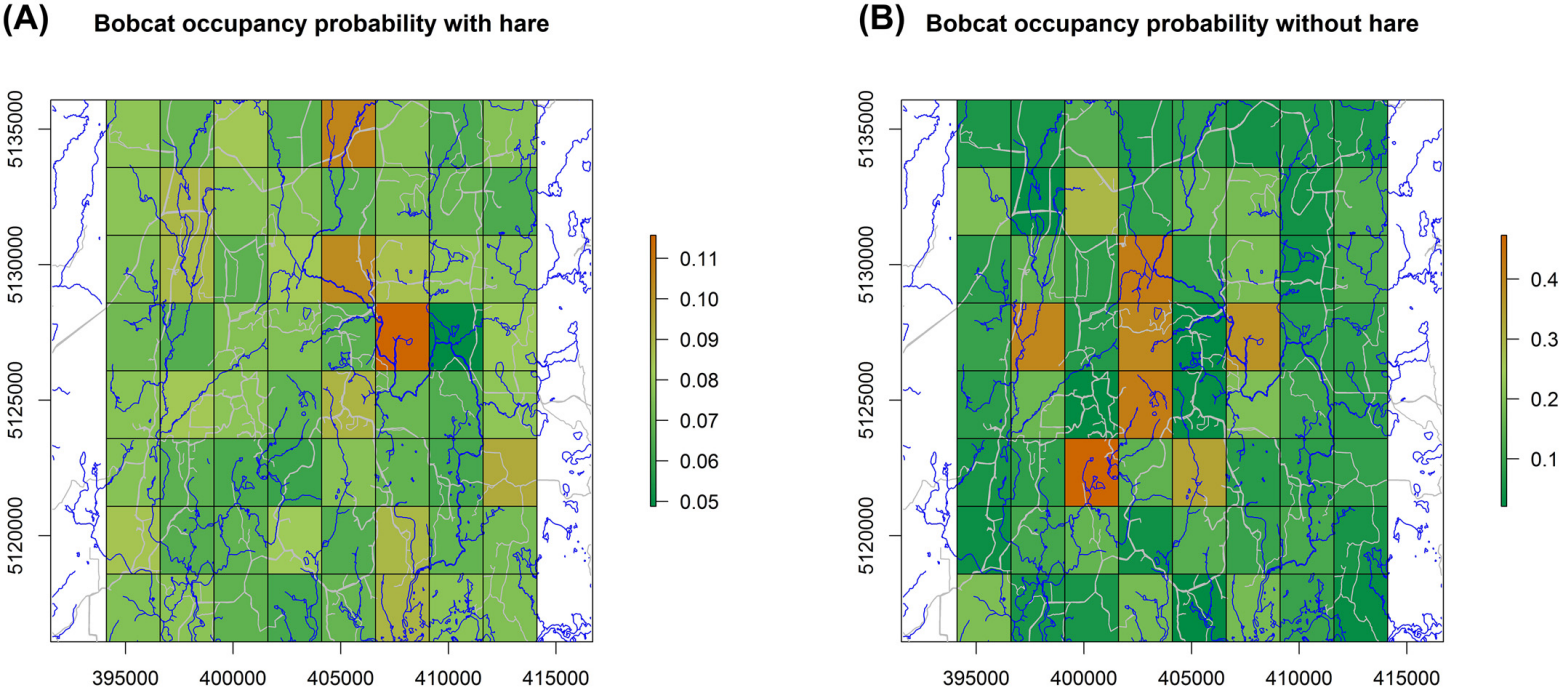
Bobcat occupancy probability when snowshoe hare are present (A) and absent (B), Upper Peninsula Michigan, December 2012–February 2013. Roads and streams are represented by grey and blue lines, respectively.

When hares were present, bobcats were present only 7.4% of the time (12.6% when hare was absent; Table 3). According to our model, and based on the observed data, there was a 94% [64%; 99%] chance that the proportion of cells occupied by bobcats was greater than the proportion of cells without bobcat when hares were absent. Conversely, when bobcat were present, hares were present only 19.6% of the time (30.5% when bobcat was absent). The probability that the proportion of cells occupied by hare was greater than the proportion of cells without hare when bobcats were present was only a 0.06 [0; 0.36]. Greater average bobcat occupancy when hare was absent appeared driven by a subset of cells in the center of our study area where occupancy probability was greater (Fig 3). The remainder of the study area had a lower bobcat occupancy probability when hare was absent, but occupancy probabilities for those cells increased when hare was present. The central area of high bobcat occupancy probability when hares were absent appeared to influence overall bobcat occupancy when hare occupancy was considered.

**Table 3:** Contingency table of the total number of cells and corresponding 95% credible intervals (summed across weeks) summarizing bobcat and snowshoe hare presence, Upper Peninsula Michigan, December 2012–February 2013.

	Hare present	Hare Absent	Total
**Bobcat present**	15.3 [111; 22]	62.9 [50; 81]	78.2 [65; 97]
**Bobcat absent**	190.7 [178; 204]	435.2 [414; 453]	625.8 [607; 639]
**Total**	205.9 [194; 219]	498.1 [485; 510]	

## Discussion

Bobcat occupancy was strongly negatively correlated with road density at the cell scale (and positively at the neighborhood scale) when hare were absent, but hare presence was the only driver of bobcat occupancy when present. In contrast, hare occupancy varied greatly over time and space, was positively correlated with grassland cover types, and possible avoidance of bobcats. Thus, bobcat and hare occupancy appeared influenced by landscape features and predator-prey relations.

### Snowshoe hare occupancy

Snowshoe hare selected for grassland cover types. Snowshoe hare presence has been associated with landcover [45], food and cover [46], habitat fragmentation [47], and predator presence and density [48][49]. Hares vacate suboptimal habitats during population declines and during population increases, use less-preferred habitats once higher-quality habitats are saturated [48]. The first cover types to be deserted are usually open or marginal habitats, then mature birch or aspen stands [46][48][50]. In contrast, hare occupancy is positively correlated with presence of dense brushy understory and conifers [40][42][43][51] as well as young forests [52]. One reason why evergreen was not selected might be that cells contained adequate amounts of this land cover such that our model would have identified it as a baseline. It is also possible that coniferous forests, while offering a dense cover, can also lack suitable amounts of preferred browse, especially during winter [51], causing hares to move among those habitat patches to more successional forests to find food.

Large scale patterns of extinction and colonization by snowshoe hares are influenced by cover characteristics, with habitat quality having greatest effect at local scales [49]. Hares exhibit daily variation in use of vegetation types, even when food and cover appear abundant in a single habitat [46][51]. Similarly, temporal variation in hare detectability arises from variation in availability for detection. For example, we demonstrated use of stations varied in response to bobcat occurrence. Consequently, including factors such as predator avoidance should be considered in future models to better understand species habitat relationships.

### Bobcat occupancy

Bobcat occupancy probabilities varied across space and time and were related to local hare presence and (when hares were absent) to road density at both local spatial scales. Local presence of snowshoe hare was the main driver for bobcat occupancy, but when hare were absent, road density influenced variation in occupancy probabilities. We demonstrated an adverse effect of roads on bobcats at the local scale. Increases in road density can negatively affect bobcat populations [53][22] through habitat loss, altering landscape structure and function, and increasing access for humans [54]. Interestingly, bobcats do not always avoid roads [55], and sometimes use them to conserve energy by avoiding walking in deeper snow [56][57]. This could explain the slight positive association we observed between bobcat occupancy probabilities and road density at the neighborhood scale. Saunders et al. suggested the potential effects of road density should be examined at appropriate scales and across different forest types and can be used to indicate the level of human disturbance [54]. Further, mature forests typically are of lower suitability for hares than successional forests [45]. As timber harvesting in our study area occurs near or adjacent to roads, subsequent greater habitat suitability for hares in these areas may facilitate selection for roads by bobcats at the coarser resolution.

Bobcat habitat selection was not only scale specific, but context specific. While road density influenced bobcat occupancy in two different ways depending on if looking at the local (i.e. cell) or larger (i.e. neighborhood) scale, this occurred only when hares were absent at the local scale. When present, hare occupancy at the local scale was the only relevant parameter to predict bobcat presence. Prey abundance and availability is an important driver of bobcat populations [58][59][60][61][62][10]. Though prey presence explained bobcat occupancy at the local scale, other factors can be more important at a larger scale (or when preys are absent). Godbois, Conner & Warren demonstrated that bobcat habitat selection at a smaller spatial scale was driven by food, whereas habitat composition was more important at coarser spatial scale, concluding that prey and refugia are important to bobcats [18]. We similarly suggest that prey presence is important to bobcats at fine spatial scales and that environmental factors become important at larger scales and when an important prey species is absent.

### Cooccurrence

In contrast to our prediction, overall bobcat occupancy was lower when hares were present. Snowshoe hare experience dramatic variation in weather, food availability, and predation pressure [51]. Hares will select habitat with denser cover when more predators are present [46][63], trading preferred browse for reduced predation risk [64][65][51]. This could explain why in our study hare occupancy appeared to be lower when bobcats were present. Avoidance of bobcats by hares at the scale of bait stations is likely to lead to lower availability of hares when bobcats are present in an area. Hare would avoid the area around the bait station when bobcats area present, but could still be present in the cell. This behavior would affect our results by lowering hare apparent occupancy probability and lowering the chances of hare detection by our cameras when bobcats were present. Consequently, the lower detection probability for hares when bobcats were present could lead to underestimation of the simultaneous presence of both species. Our results support this hypothesis as hares actively avoided bobcats.

Other factors could influence bobcat occupancy, including alternate prey. In Massachusetts, white-tailed deer were used more by bobcats than snowshoe hares, despite hares being more abundant [56]. In this same study, once a deer had been killed, due to its large body size bobcats would apparently feed for up to 3–4 days. Finally, bobcats could stay in areas of their home range offering better refugia, only occasionally leaving them for hunting in cells where preys are more readily available. Similarly, space use of lynx in Alberta was not related to snowshoe hare density; rather, lynx met their food requirements by switching to alternate prey including carrion.

Several improvements for our approach are possible. Currently, the conclusions drawn from our modeling approach and methods are limited by several factors. First, the current resolution of our sampling design limits our inferences of the relationship between bobcat and hare presence. Snowshoe hare home range sizes have been report to be up to 6 ha [51], while our study design resolution (i.e. cell size) correspond to 625 ha. Therefore, the importance of potential relationships, among hare presence, bobcat presence, and habitat covariates summarized at this resolution might not accurately reflect the effect of these covariates at the scale of snowshoe hare’s home range. Moreover, since we only have one bait station per cell, our results rely on the assumption that each station provides a valid representation of the processes occurring in its corresponding cell. Increasing our sampling effort by augmenting the total number of bait stations should help regarding these issues. Moreover, setting several stations in each cell could facilitate i) achieving more accurate estimates of cell occupancy and ii) estimating variation in hare use of bait stations within weeks in response to bobcat presence. However, increasing the number of bait station could also cause another problem. One essential feature of this design is that each station should accurately sample bobcat occupancy in its corresponding cell, i.e. it should not attract individuals from neighboring cells. In this context, the widely accepted use of bait stations for carnivore studies represents a challenge for habitat use under the occupancy modeling paradigm. However, this approach can prove to be especially useful in the case of rare or hard-to-detect species [66] and has been proven to be a viable method for estimating occupancy at both local and landscape scales for at least some carnivores [67]. Any subsequent design should account for the risk of affecting perceived habitat use (via the attraction of individual from other cells) by optimizing the bait station density against their attractiveness. To our knowledge, there is not good data on bobcat detection distances of lures. However, it has been suggested black bears could likely detect lures up to about 135m away [68], and we have circumstantial evidences of bobcats not detecting our scented and baited traps from just a few meters away, depending on the wind direction. For all our carnivore trapping, we have not seen evidence of attraction of more than several meters, and this confirms why trap/site location is so important. Finally, future extensions of this model could benefit from modeling hare detection probability conditionally on bobcat presence, or conversely bobcat detectability in function of hare presence [34]. This could improve our understanding of the relationship between bobcat (or hare) presence and hare (or bobcat) presence and detectability. One final consideration could be to directly account for occupancy (presence/absence over weeks) and use (variation in occupancy among days within a week) to further our understanding of the dynamic use of their habitat by our target species [69].

Habitat use by bobcats [52] and other carnivore species is hierarchical [17][7]. We identified some factors driving bobcat and snowshoe hare occupancies and the importance of understanding interactions among these factors across multiple spatial scales. Importantly, we demonstrated that the effects of those factors on bobcat occupancy were context specific to predation and the presence of a primary prey species. While our model would benefit by the addition of more dynamic covariates to explain weekly variations in bobcat and snowshoe hare occupancy, we demonstrate how scale, environment, and prey presence can interact to explain carnivore occupancy. When trying to understand the factors underlying carnivore distributions, we recommend addressing landscape attributes in addition to factors controlling prey species dynamics and at which spatial scales those factors are acting.

## Supporting Information

S1 Table Range of the covariates used in the selected bobcat-snowshoe hare hierarchical co-occurrence model in Upper Peninsula Michigan, during December 2012—February 2013. (DOCX)

S1 File Winbugs code for the selected bobcat-hare cooccurrence model (Upper Peninsula Michigan, December 2012–February 2013). (DOCX)

S1 Dataset R workspace containing the data used in the selected bobcat-snowshoe hare hierarchical co-occurrence model in Upper Peninsula Michigan, during December 2012—February 2013. (RDATA)

S1 Fig Goodness-of-fit for model with bobcat and snowshoe hare detection probabilities constant over space and time. (JPG)

S2 Fig Goodness-of-fit for model with cell-specific bobcat detection probability and cell-week-specific hare bobcat detection probability. (JPG)
