# Supplementary material for: Effects of Prey Presence and Scale on Bobcat Resource Selection during Winter
Source: PLoS One. 2015 Nov 18;10(11):e0143347. doi: 10.1371/journal.pone.0143347 (PMC4651546; doi:10.1371/journal.pone.0143347)
Supplement: S1 Table — (DOCX) [file pone.0143347.s001.docx]

**S1 Table.** Range of the covariates used in the selected bobcat-snowshoe hare hierarchical co-occurrence model in Upper Peninsula Michigan, during December 2012 - February 2013.

| Covariate | Scale | Range |
| --- | --- | --- |
| Road density | Cell | [-1.48 ; 3.05] |
| Road density | Neighborhood | [-2.49 ; 2.62] |
| Water density | Cell | [-1.72 ; 2.74] |
| Water density | Neighborhood | [-1.28 ; 3.17] |
| Distance to water |  | [-0.90 ; 2.98] |
| DEA | Cell | [-1.61 ; 2.63] |
| Aspen | Cell | [-1.62 ; 2.76] |
| Mixed | Cell | [-1.36 ; 3.11] |
| Shrub | Cell | [-0.68 ; 3.33] |
| WW | Cell | [-1.73 ; 3.76] |
| Unsuitable | Cell | [-0.75 ; 4.04] |
| Evergreen | Cell | [-1.05 ; 3.19] |
| Grassland | Cell | [-0.91 ; 3.58] |
| EHW | Cell | [-0.58 ; 5.11] |
