# Supplementary material for: Effects of Prey Presence and Scale on Bobcat Resource Selection during Winter
Source: PLoS One. 2015 Nov 18;10(11):e0143347. doi: 10.1371/journal.pone.0143347 (PMC4651546; doi:10.1371/journal.pone.0143347)
Supplement: S1 File — (DOCX) [file pone.0143347.s002.docx]

**S1 File**. Winbugs code for the selected bobcat-hare cooccurrence model (Upper Peninsula Michigan, December 2012–February 2013).

| model{  ### ECOLOGICAL PROCESS ###  # Priors for bobcat  tau.eps.bobcat.with.hare <-1/(sd.eps.bobcat.with.hare*sd.eps.bobcat.with.hare)  sd.eps.bobcat.with.hare ~ dgamma(0.01,0.01)  intercept.bobcat.with.hare ~ dflat()  alpha.road.density.with.hare ~ dnorm(0,0.0001)  alpha.hydro.density.with.hare ~ dnorm(0,0.0001)  alpha.hydro.dist.with.hare ~ dnorm(0,0.0001)  beta.road.density.with.hare ~ dnorm(0,0.0001)  beta.hydro.density.with.hare ~ dnorm(0,0.0001)  tau.eps.bobcat.without.hare <-1/(sd.eps.bobcat.without.hare*sd.eps.bobcat.without.hare)  sd.eps.bobcat.without.hare ~ dgamma(0.01,0.01)  intercept.bobcat.without.hare ~ dflat()  alpha.road.density.without.hare ~ dnorm(0,0.0001)  alpha.hydro.density.without.hare ~ dnorm(0,0.0001)  alpha.hydro.dist.without.hare ~ dnorm(0,0.0001)  beta.road.density.without.hare ~ dnorm(0,0.0001)  beta.hydro.density.without.hare ~ dnorm(0,0.0001)  # Priors for hare  intercept.hare ~ dflat()  m.DEA ~ dnorm(0,0.01)  m.evergreen ~ dnorm(0,0.01)  m.mixed ~ dnorm(0,0.01)  m.shrub ~ dnorm(0,0.01)  m.grass ~ dnorm(0,0.01)  m.WW ~ dnorm(0,0.01)  m.EHW ~ dnorm(0,0.01)  m.unsuitable ~ dnorm(0,0.01)  m.aspen ~ dnorm(0,0.01)  tau.eps.hare <-1/(sd.eps.hare*sd.eps.hare)  sd.eps.hare ~ dgamma(0.01,0.01)  # Priors for model selection  for (m in 1:19){  mod.sel[m]~dbern(0.5)  }  for ( i in 1:nsites){  # Site-specific covariates  cell.with.hare[i] <- mod.sel[1]*alpha.road.density.with.hare*road.cell[i] + mod.sel[2]*alpha.hydro.density.with.hare*hydro.cell[i]  neigh.with.hare[i] <- mod.sel[3]*beta.road.density.with.hare*road.neigh[i] + mod.sel[4]*beta.hydro.density.with.hare*hydro.neigh[i]  cell.without.hare[i] <- mod.sel[5]*alpha.road.density.without.hare*road.cell[i] + mod.sel[6]*alpha.hydro.density.without.hare*hydro.cell[i]  neigh.without.hare[i] <- mod.sel[7]*beta.road.density.without.hare*road.neigh[i] + mod.sel[8]*beta.hydro.density.without.hare*hydro.neigh[i]  cover[i]<- mod.sel[9]*m.DEA*cov.data[i,1] + mod.sel[10]*m.mixed*cov.data[i,3] + mod.sel[11]*m.shrub*cov.data[i,4] + mod.sel[12]*m.WW*cov.data[i,6] + mod.sel[13]*m.unsuitable*cov.data[i,8] + mod.sel[14]*m.aspen*cov.data[i,9] + mod.sel[15]*m.evergreen*cov.data[i,2] + mod.sel[16]*m.grass*cov.data[i,5] + mod.sel[17]*m.EHW*cov.data[i,7]  for ( t in 1:nweeks){  # Bobcat occupancy conditional on hare presence  Z.bobcat[i,t]~dbern(mu.bobcat[i,t])  mu.bobcat[i,t]<- mu.bobcat.with.hare[i,t]*Z.hare[i,t] + mu.bobcat.without.hare[i,t]*(1-Z.hare[i,t])  logit(mu.bobcat.with.hare[i,t])<-max(min(20,logit.mu.bobcat.with.hare[i,t]),-20)  logit.mu.bobcat.with.hare[i,t] <- intercept.bobcat.with.hare + epsilon.bobcat.with.hare[i,t] + cell.with.hare[i] + neigh.with.hare[i] + mod.sel[18]*alpha.hydro.dist.with.hare*dist.from.hydro[i]  logit(mu.bobcat.without.hare[i,t])<-max(min(20,logit.mu.bobcat.without.hare[i,t]),-20)  logit.mu.bobcat.without.hare[i,t] <- intercept.bobcat.without.hare + epsilon.bobcat.without.hare[i,t] + cell.without.hare[i] + neigh.without.hare[i] + mod.sel[19]*alpha.hydro.dist.without.hare*dist.from.hydro[i]  # Hare occupancy  Z.hare[i,t]~dbern(mu.hare[i,t])  logit(mu.hare[i,t])<-max(min(20,logit.mu.hare[i,t]),-20)  logit.mu.hare[i,t] <- intercept.hare + epsilon.hare[i,t] + cover[i]  # Over-dispersion priors  epsilon.bobcat.with.hare[i,t] ~ dnorm(0,tau.eps.bobcat.with.hare)  epsilon.bobcat.without.hare[i,t] ~ dnorm(0,tau.eps.bobcat.without.hare)  epsilon.hare[i,t] ~ dnorm(0,tau.eps.hare)  }  }  ### OBSERVATION PROCESS ###  for ( i in 1:nsites){  p.bobcat[i] ~ dunif(0,1)  for ( t in 1:nweeks){  p.hare[i,t] ~ dunif(0,1)  }  }  for( k in 1:nrecords) {  Y.bobcat[k] ~ dbin(proba.bobcat[k],occasion[k])  proba.bobcat[k]<-p.bobcat[site[k]]*Z.bobcat[site[k],week[k]]  Y.hare[k] ~ dbin(proba.hare[k],occasion[k])  proba.hare[k]<-p.hare[site[k],week[k]]*Z.hare[site[k],week[k]]  }  ### Stats  # Bayesian analysis of 2×2 contingency table  for ( i in 1:nsites){  for ( t in 1:nweeks){  BpHp[i,t]<-Z.bobcat[i,t]*Z.hare[i,t]  BpHa[i,t]<-Z.bobcat[i,t]*(1-Z.hare[i,t])  BaHp[i,t]<-(1-Z.bobcat[i,t])*Z.hare[i,t]  BaHa[i,t]<-(1-Z.bobcat[i,t])*(1-Z.hare[i,t])  }  }  BpHp.obs<-sum(BpHp[,])  BpHa.obs<-sum(BpHa[,])  BaHp.obs<-sum(BaHp[,])  BaHa.obs<-sum(BaHa[,])  Bp<-BpHp.obs+BpHa.obs  Ba<-BaHp.obs+BaHa.obs  Hp<-BpHp.obs+BaHp.obs  Ha<-BpHa.obs+BaHa.obs  Tot<-Bp+Ba    BpHp.exp<-Bp*Hp/Tot  BpHa.exp<-Bp*Ha/Tot  BaHp.exp<-Ba*Hp/Tot  BaHa.exp<-Ba*Ha/Tot    # cf http://lingpipe-blog.com/2009/10/13/bayesian-counterpart-to-fisher-exact-test-on-contingency-tables/  # http://andrewgelman.com/2009/10/13/what_is_the_bay/    # DATA SIMULATION  scaleBp1A <- BpHp.obs+1  scaleBp1B <- (Bp-BpHp.obs)+1  scaleBa1A <- BaHp.obs+1  scaleBa1B <- (Ba-BaHp.obs)+1    scaleHp1A <- BpHp.obs+1  scaleHp1B <- (Hp-BpHp.obs)+1  scaleHa1A <- BpHa.obs+1  scaleHa1B <- (Ha-BpHa.obs)+1    scaleBp2A <- BpHa.obs+1  scaleBp2B <- (Bp-BpHa.obs)+1  scaleBa2A <- BaHa.obs+1  scaleBa2B <- (Ba-BaHa.obs)+1  scaleHp2A <- BaHp.obs+1  scaleHp2B <- (Hp-BaHp.obs)+1  scaleHa2A <- BaHa.obs+1  scaleHa2B <- (Ha-BaHa.obs)+1  for( it in 1:10000){  # simulations    thetaBp1[it] ~ dbeta(scaleBp1A, scaleBp1B)  thetaBa1[it] ~ dbeta(scaleBa1A, scaleBa1B)    thetaHp1[it] ~ dbeta(scaleHp1A, scaleHp1B)  thetaHa1[it] ~ dbeta(scaleHa1A, scaleHa1B)    thetaBp2[it] ~ dbeta(scaleBp2A, scaleBp2B)  thetaBa2[it] ~ dbeta(scaleBa2A, scaleBa2B)  thetaHp2[it] ~ dbeta(scaleHp2A, scaleHp2B)  thetaHa2[it] ~ dbeta(scaleHa2A, scaleHa2B)  diffB1[it] <- thetaBp1[it]-thetaBa1[it] # simulated diffs  diffH1[it] <- thetaHp1[it]-thetaHa1[it] # simulated diffs    probB1.tmp[it]<-1-step(-diffB1[it])  probH1.tmp[it]<-1-step(-diffH1[it])    diffB2[it] <- thetaBp2[it]-thetaBa2[it] # simulated diffs  diffH2[it] <- thetaHp2[it]-thetaHa2[it] # simulated diffs  probB2.tmp[it]<-1-step(-diffB2[it])  probH2.tmp[it]<-1-step(-diffH2[it])  }  probB1 <- mean(probB1.tmp[]) # Probability Bp higher than Ba when Hp  probH1 <- mean(probH1.tmp[]) # Probability Hp higher than Ha when Bp  probB2 <- mean(probB2.tmp[]) # Probability Bp higher than Ba when Ha  probH2 <- mean(probH2.tmp[]) # Probability Hp higher than Ha when Ba  # GOF for bobcat  for( k in 1:nrecords) {    # Compute fit statistics for observed data  eval.bobcat[k]<-proba.bobcat[k]*occasion[k]  E.bobcat[k]<- pow((Y.bobcat[k]-eval.bobcat[k]),2)/(eval.bobcat[k]+0.5)    # Generate replicate data and compute fit stats for them  Y.bobcat.new[k] ~ dbin(proba.bobcat[k],occasion[k])  E.bobcat.new[k]<- pow((Y.bobcat.new[k]-eval.bobcat[k]),2)/(eval.bobcat[k]+0.5)  }    fit.bobcat<-sum(E.bobcat[])  fit.bobcat.new<-sum(E.bobcat.new[])  # GOF for hare  for( k in 1:nrecords) {  # Compute fit statistics for observed data  eval.hare[k]<-proba.hare[k]*occasion[k]  E.hare[k]<- pow((Y.hare[k]-eval.hare[k]),2)/(eval.hare[k]+0.5)  # Generate replicate data and compute fit stats for them  Y.hare.new[k] ~ dbin(proba.hare[k],occasion[k])  E.hare.new[k]<- pow((Y.hare.new[k]-eval.hare[k]),2)/(eval.hare[k]+0.5)  }  fit.hare<-sum(E.hare[])  fit.hare.new<-sum(E.hare.new[])  } |
| --- |
