# Supplementary figures and images for: Effects of Prey Presence and Scale on Bobcat Resource Selection during Winter
Source: PLoS One. 2015 Nov 18;10(11):e0143347. doi: 10.1371/journal.pone.0143347 (PMC4651546; doi:10.1371/journal.pone.0143347)

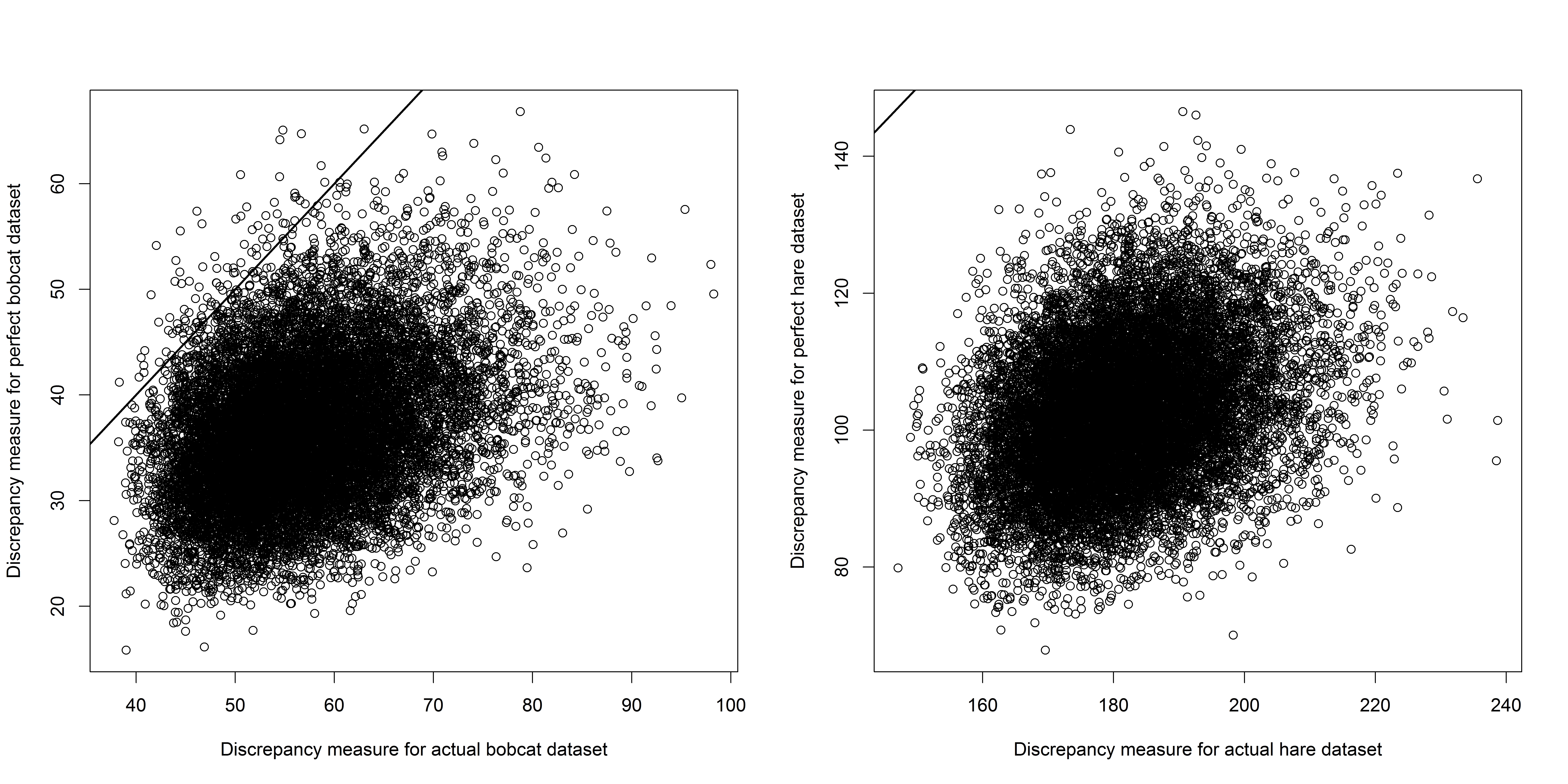

Supplement: S1 Fig — (JPG) [file pone.0143347.s004.jpg]

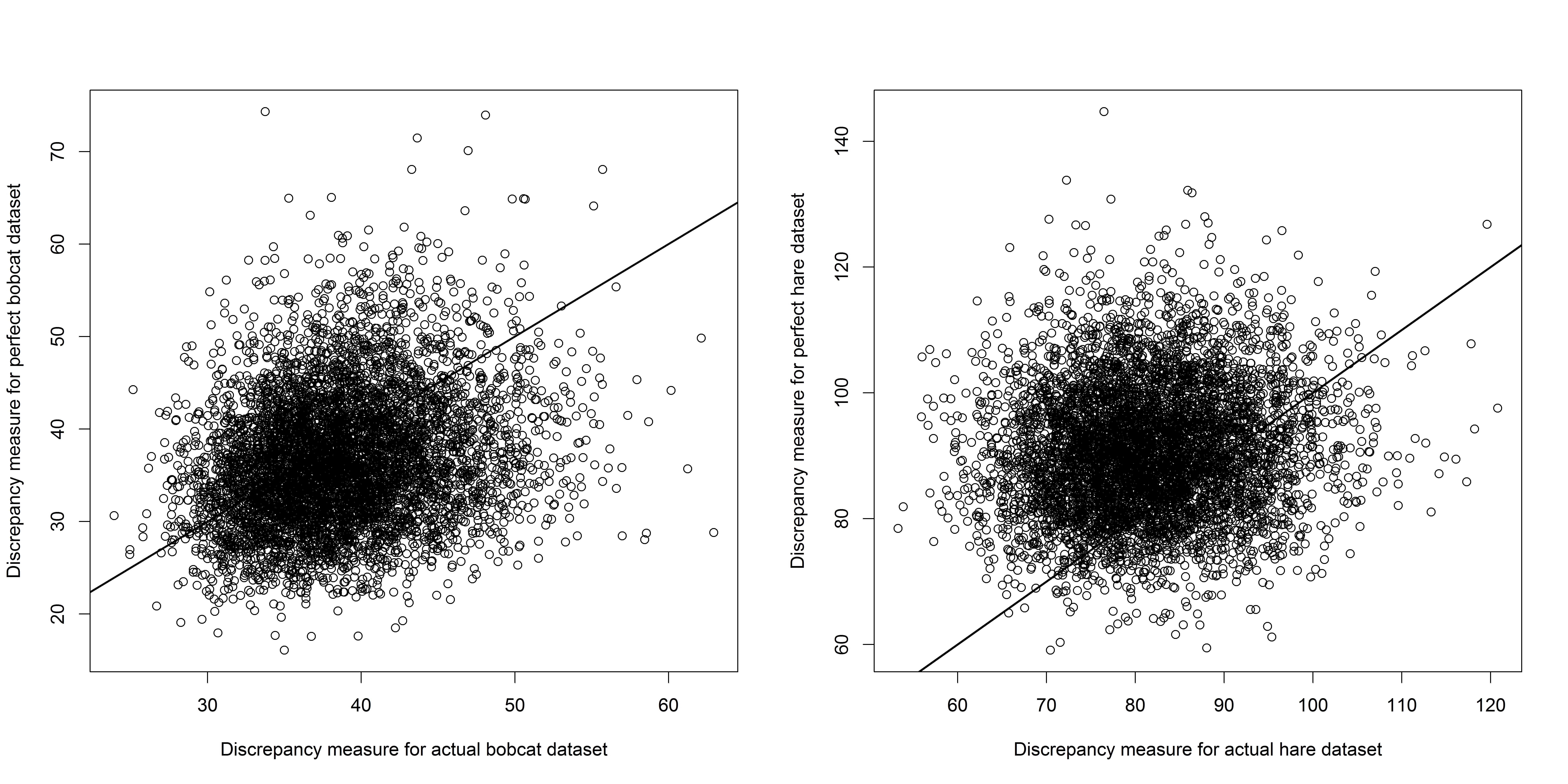

Supplement: S2 Fig — (JPG) [file pone.0143347.s005.jpg]
